# HSP90/AXL/eIF4E-regulated unfolded protein response as an acquired vulnerability in drug-resistant *KRAS*-mutant lung cancer

**DOI:** 10.1038/s41389-019-0158-7

**Published:** 2019-08-20

**Authors:** Haitang Yang, Shun-Qing Liang, Duo Xu, Zhang Yang, Thomas M. Marti, Yanyun Gao, Gregor J. Kocher, Heng Zhao, Ralph A. Schmid, Ren-Wang Peng

**Affiliations:** 1 Department of General Thoracic Surgery, Inselspital, Bern University Hospital, Bern, Switzerland; 2 Department for BioMedical Research (DBMR), University of Bern, Bern, Switzerland; 3 Department of Thoracic Surgery, Shanghai Chest Hospital, Shanghai Jiao Tong University, 200030 Shanghai, China; 4 Present Address: University of Massachusetts Medical School, Worcester, MA 01605 USA

**Keywords:** Non-small-cell lung cancer, Oncogenes, Cancer therapeutic resistance

## Abstract

Drug resistance and tumor heterogeneity are formidable challenges in cancer medicine, which is particularly relevant for *KRAS*-mutant cancers, the epitome of malignant tumors recalcitrant to targeted therapy efforts and first-line chemotherapy. In this study, we delineate that *KRAS*-mutant lung cancer cells resistant to pemetrexed (MTA) and anti-MEK drug trametinib acquire an exquisite dependency on endoplasmic reticulum (ER) stress signaling, rendering resistant cancer cells selectively susceptible to blockage of HSP90, the receptor tyrosine kinase AXL, the eukaryotic translation initiation factor 4E (eIF4E), and the unfolded protein response (UPR). Mechanistically, acquisition of drug resistance enables *KRAS*-mutant lung cancer cells to bypass canonical KRAS effectors but entail hyperactive AXL/eIF4E, increased protein turnover in the ER, and adaptive activation of an ER stress-relief UPR survival pathway whose integrity is maintained by HSP90. Notably, the unique dependency and sensitivity induced by drug resistance are applicable to *KRAS*-mutant lung cancer cells undergoing de novo intratumor heterogeneity. In line with these findings, HSP90 inhibitors synergistically enhance antitumor effects of MTA and trametinib, validating a rational combination strategy to treat *KRAS*-mutant lung cancer. Collectively, these results uncover collateral vulnerabilities co-occurring with drug resistance and tumor heterogeneity, informing novel therapeutic avenues for *KRAS*-mutant lung cancer.

## Introduction

Resistance to therapeutics and tumor heterogeneity inevitably limit clinical efficacy of cancer treatment, which is particularly relevant for *KRAS*-mutant cancers that are the most common type of human malignancies defined by genetic alterations and, ironically, the largest subset of tumors that cannot be effectively targeted by currently available therapeutics^1^. Oncogenic mutant KRAS is associated with striking heterogeneity^2^, heightened resistance to first-line chemotherapy^3,4^, and has proven difficult to pharmacologically target directly^5^, as have attempts to block synthetic lethal interactions with a mutant *KRAS* allele that have met with only limited success^6^.

Innovative efforts to inhibit *KRAS*-dependent tumor growth by extinguishing KRAS downstream signaling pathways have led to the important observation that suppressing the mitogen-activated protein kinase (MAPK) cascade RAF/MEK/ERK is necessary but insufficient^7^. Consequently, highly selective RAF, MEK, and ERK inhibitors only show marginal antitumor activity in *KRAS*-mutant tumors^8^. A major cause for the inefficiency of MAPK inhibitors, such as the clinically approved anti-MEK drug trametinib^9^, is the rapid and inevitable development of resistant diseases. The transient or short-lived nature of trametinib has been attributed to treatment-induced repression of negative feedback loops that reactivates the MAPK pathway via various compensatory mechanisms^10–13^. Nevertheless, albeit improved effects of MEK inhibitors by blocking compensatory MAPK reactivation, resistant tumors on the combination treatment still evolve^14^, suggesting the existence of other uncharacterized mechanisms of adaptive resistance.

In this study, we investigated the molecular mechanisms underlying adaptive resistance to currently available cancer drugs in *KRAS*-mutant lung cancer. We showed that HSP90/AXL/eIF4E-regulated endoplasmic reticulum (ER) stress response, or the unfolded protein response (UPR), represents an acquired dependency and confers a selective vulnerability in drug-resistant *KRAS*-mutant lung cancer cells that drug-naïve parental cells lack. We further demonstrated that this collateral sensitivity induced by drug resistance also applies to *KRAS*-mutant lung cancer cells that have undergone de novo intratumor heterogeneity. These results uncover tumor vulnerabilities that can be therapeutically exploited to overcome drug resistance and tumor heterogeneity, providing a new rationale for combination therapies to combat aggressive and difficult-to-treat *KRAS*-mutant lung cancer.

## Results

### A shared mechanism of resistance to MTA and trametinib in *KRAS*-mutant lung cancer

**Fig. 1:**
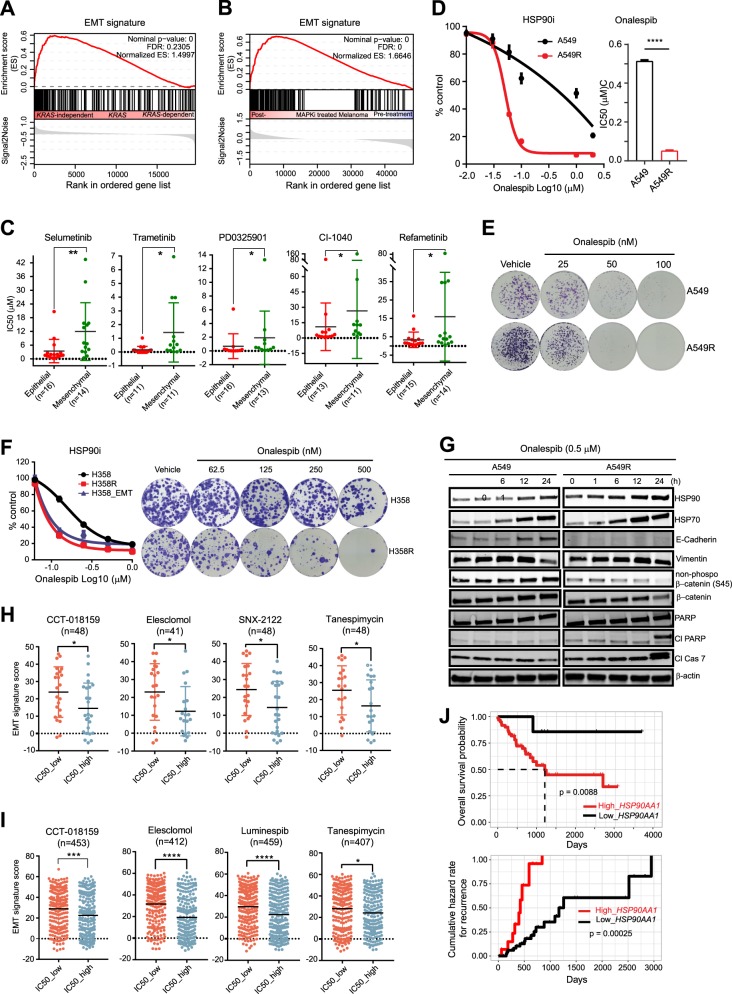
HSP90 inhibitors selectively target *KRAS*-mutant lung cancer cells resistant to chemo and anti-MEK drugs. **a**, **b** Gene set enrichment analysis (GSEA) of *KRAS*-mutant cancer cell lines (**a**) and matched tumor samples (pre- and post-treatment with MAPK (BRAF + MEK) inhibitors of patients with *BRAF*-mutant melanoma (**b**) revealed significant enrichment of EMT gene signatures in *KRAS*-independent cell lines (**a**) and in residual melanoma resistant to and recurrent after MAPK inhibition (**b**). The Gene Expression Omnibus (GEO) dataset GSE15126 (**a**) and GSE65185 (**b**) were used for GSEA. **c** Mesenchymal phenotype is inversely associated with sensitivity to MEK inhibitors in *KRAS*-mutant cancer cell lines. Dichotomization of epithelial and mesenchymal subgroups is based on an EMT gene signature defined by the GSE15126 dataset. *p < 0.05, **p < 0.01. **d**, **e** Dose–response curves (**d**) and clonogenic assay (**e**) of A549R (mesenchymal) and A549 (epithelial) cells using the HSP90 inhibitor onalespib. The half-maximal inhibitory concentration (IC_50_) is shown to the right (**d**). ****p < 0.0001, unpaired two-sided *t-*test. **f** Dose–response curves (left) and clonogenic assay (right) of mesenchymal (H358R, H358_EMT) and parental H358 cells using the HSP90 inhibitor onalespib. **g** Immunoblots of A549 and A549R cells treated with onalespib (0.5 µM) for the indicated time. **h**, **i** Association of the sensitivity to HSP90 inhibitors with mesenchymal cell states in lung adenocarcinoma (**h**) and solid cancer cell lines (**i**). Gene expression data and IC_50_ values are downloaded from the Cancer Cell Line Encyclopedia and the Genomics of Drug Sensitivity in Cancer, respectively. Dichotomization of IC_50__low and IC_50__high is based on the median IC_50_ value. *p < 0.05, **p < 0.01, ***p < 0.001, ****p < 0.0001. **j** Kaplan–Meier analysis of *KRAS*-mutant lung cancer based on *HSP90AA1* expression. Patients with high- (in red) or low-*HSP90AA1* (in black) were stratified by optimal cutoff value of the *HSP90AA1* across all patients using the surv_cutpoint function in the R “maxstat” package. Overall survival and cumulative hazard rates are analyzed and plotted using the R “survival” and “survminer” package. The *p* value is calculated by the log-rank test

To delineate cellular and molecular mechanisms that underpin drug resistance in *KRAS*-mutant lung cancer, we modeled adaptive resistance by step-wisely and chronically treating A549 and H358 cells with chemotherapy drug pemetrexed (MTA) whereby resistant populations (A549R and H358R) persisted after the treatment. A549R and H358R cells showed cross-resistance to trametinib, indicating a shared mechanism of resistance to the two cancer drugs (Supplementary Fig. 1A–C). Drug-resistant A549R and H358R underwent profound changes in E-Cadherin or/and Vimentin compared to the parental cells (Supplementary Fig. 1A–C), which is characteristic of cell state switch through an epithelial-to-mesenchymal transition (EMT). Alike, de novo enforcement of mesenchymal reprogramming by treating A549 and H358 with transforming growth factor (TGF-β1) that fueled dedifferentiated subpopulations (A549_EMT, H358_EMT) co-opted acquired resistance to MTA and trametinib (Supplementary Fig. 1D, E). Supporting our results, analyzing transcriptomic data of *KRAS*-mutant cancer cells^2^ and matched tumors (pre- and post-treatment with BRAF and MEK inhibitors) from patients with *BRAF*-mutant melanoma^15^ revealed that EMT gene signatures are significantly enriched in *KRAS*-mutant cancer cells resistant to *KRAS* suppression (*KRAS*-independent) and in residual melanoma tumors refractory to and recurrent after MAPK (BRAF + MEK) inhibition (Fig. 1a, b). Importantly, the *KRAS*-mutant cancer cells independent of KRAS function, driven by mesenchymal reprogramming (high EMT gene signatures)^2,16^, exhibited significantly greater resistance to various MEK inhibitors (Fig. 1c). Mining the data from The Cancer Genome Atlas (TCGA) showed that EMT is positively correlated with tumor progression and predicts poor prognosis in *KRAS*-mutant lung cancer (Supplementary Fig. 1F, G). These results underscore the clinical relevance of our experimental models and uncover a common principle through which *KRAS*-mutant lung cancer cells acquire resistance to MTA and trametinib.

### HSP90 dictates adaptive resistance to MTA and trametinib in *KRAS*-mutant lung cancer cells

To identify cellular processes that represent selective dependencies of drug-resistant cells, we performed drug screening using small molecules targeting various pathways (Supplementary Table 1). Among the compounds tested, only onalespib and R428, inhibitors of the molecular chaperone HSP90 and the receptor tyrosine kinase (RTK) AXL, differentially impaired the resistant versus parental cells, causing over 10- and 3-fold reduction of the half-maximal inhibitory concentration (IC_50_) in A549R compared to A549, respectively (Fig. 1d, e; Supplementary Fig. 2A, B). In contrast, other compounds, including inhibitors of the RTKs (FGFR, EGFR) and KRAS effectors (PI3K, STAT3) previously reported to mediate resistance to MEK inhibitors^12,17–20^ showed no differential effect on A549R and A549. Similarly, PU-WS13, a selective inhibitor of the ER-resident HSP90 isoform GRP94 (ref. ^21^), was equal for A549R and A549 (Supplementary Fig. 2A), indicating that the selective effect of onalespib on A549R is due to the inhibition of the cytosolic HSP90. Further analyses using other HSP90 inhibitors (luminespib, ganetespib) and drug-resistant cells (A549_EMT, H358R, and H358_EMT) led to similar findings (Fig. 1f; Supplementary Fig. 2B–G).

HSP90 is moderately overexpressed in drug-resistant relative to parental cells, but HSP90 inhibition with onalespib (0.5 µM) selectively induced apoptosis in resistant cells only, marked by dramatic increase of cleaved poly(ADP-ribose) polymerase (Cl PARP) and caspase 7 (Cl Cas7) at 24 h (Fig. 1g; Supplementary Figs. 2E and 3A). Notably, onalespib blocked EMT in A549 and H358 cells, evidenced by upregulated E-Cad and non-phospho-β-catenin, but failed to do so in drug-resistant cells (Fig. 1g; Supplementary Figs. 2E and 3A), indicating that an intact HSP90 is essential for drug-resistant mesenchymal cell state. In agreement, the EMT gene signature is a biomarker that positively determines the sensitivity of *KRAS*-mutant and pan-solid cancer cells to various HSP90 inhibitors (Fig. 1h, i) and high levels of *HSP90AA1* (HSP90) are associated with tumor progression, poor survival, and high recurrence rates in *KRAS*-mutant and pan-cancers (Fig. 1j; Supplementary Fig. 3B–E). These results identify HSP90 as a particular dependency of *KRAS*-mutant lung cancer cells acquiring resistance to MTA and trametinib.

### Hyperactive AXL/eIF4E defines drug-resistant *KRAS*-mutant lung cancer cells

**Fig. 2:**
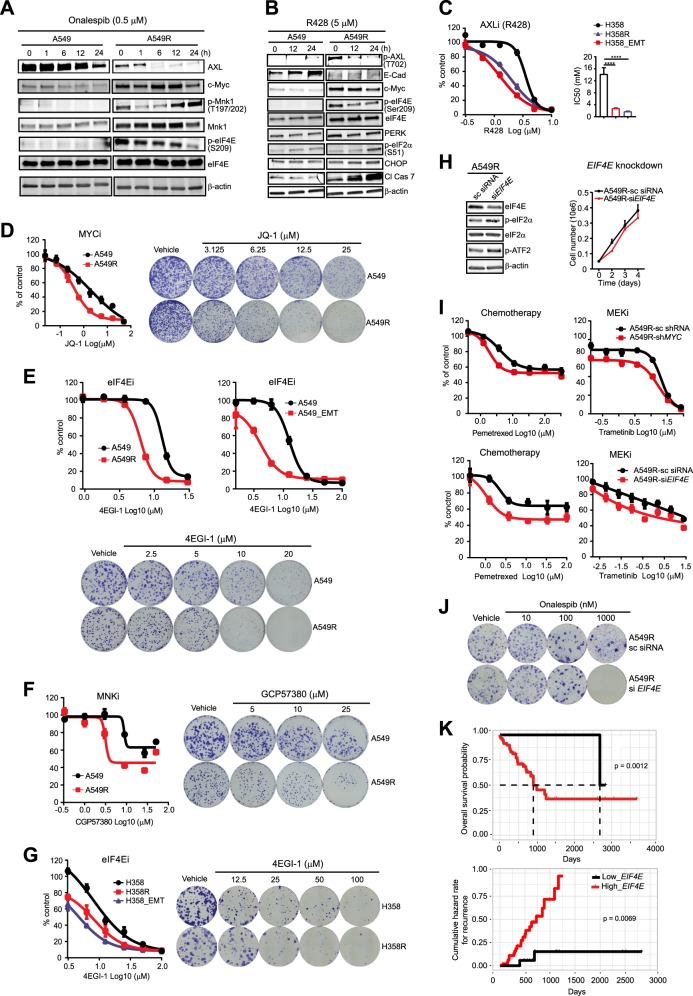
Hyperactive AXL/MYC/eIF4E defines drug-resistant *KRAS*-mutant lung cancer cells. **a**, **b** Immunoblots of A549 and A549R cells treated with onalespib (**a**) or R428 (**b**) for the indicated time. **c–g** Dose–response curves to R428 (**c**), JQ-1 (**d**), 4EGI-1 (**e**, **g**), and CGP57380 (**f**) assayed on resistant (A549R, A549_EMT, H358R, H358_EMT) and parental (A549, H358) cells. Representative results of clonogenic assays are shown to the right. **h** Immunoblots of A549R cells transfected with *EIF4E*-specific and control siRNAs. Growth curves of the transfected cells are shown to the right. **i** Dose–response curves to pemetrexed (MTA) and trametinib determined in A549R cells transfected with *MYC- or EIF4E*-specific and control shRNAs or siRNAs. **j** Clonogenic assay of A549R cells expressing *EIF4E-*specific or control siRNAs after treatment with the indicated doses of onalespib. **k** Kaplan–Meier analysis of a TCGA cohort of patients with *KRAS*-mutant lung cancer. Stratification of patients into high_ *EIF4E* (in red) and low_ *EIF4E* (in black) is based on the optimal cutoff value of *EIF4E* transcripts across all patients by using the surv_cutpoint function in R “maxstat” package. Overall survival curves and cumulative hazard rates were analyzed and plotted by using R “survival” and “survminer” packages. The *p* value is calculated using the log-rank test

Next, we investigated signaling events that underlie HSP90 requirement in drug-resistant *KRAS*-mutant lung cancer cells. A549R and A549 cells displayed similar basal activities in RAF/MEK/ERK and PI3K/AKT/mTOR pathways, as were the cells treated with onalespib (Supplementary Fig. 3F), matching the results of drug screening using inhibitors of these pathways (Supplementary Fig. 2A). In contrast, the drug-resistant cells (A549R, H358R, and H358_EMT) overexpressed MYC (also known as c-MYC), MNK1 (MAP kinase-interacting serine/threonine-protein kinase 1), and eIF4E, and showed overwhelming phosphorylation (activation) in AXL (p-AXL), MNK1 (p-MNK1), and eIF4E (p-eIF4E). However, onalespib (0.5 µM) acutely reduced AXL, MYC, and p-eIF4E whereas the total eIF4E was not affected, as did R428 (Fig. 2a, b; Supplementary Fig. 3A). Notably, like onalespib, R428 induced apoptosis (Cl Cas7) in A549R only, whereas it blocked EMT by inducing a mesenchymal-to-epithelial transition (MET) in A549 (Fig. 2b). This effect was faithfully translated to an increased toxicity of R428 on drug-resistant cells (Fig. 2c). In agreement with other studies^22,23^, we demonstrated that AXL complexed with HSP90 (Supplementary Fig. 4A) and that *AXL* gene and protein levels were positively correlated with EMT (Supplementary Fig. 4B–D). Importantly, the *AXL* mRNA level is a predictive marker for poor prognosis in lung cancer patients (Supplementary Fig. 4E, F).

Small-molecule inhibitors of MYC (JQ-1), MNK1 (CGP57380), and eIF4E/eIF4G complex (4EGI-1) more strongly suppressed the resistant than parental cells (Fig. 2d–g), as did *MYC and EIF4E* knockdown that barely affected cell proliferation but markedly reduced resistance to MTA and trametinib and augmented the sensitivity to onalespib (Fig. 2h–j; Supplementary Fig. 4G). Further, the *MYC* expression is positively associated with *AXL*, *EIF4E*, and EMT signatures (Supplementary Fig. 4H) and both mRNA and protein levels of the *EIF4E* are of prognostic significance in *KRAS*-mutant lung cancer, lung adenocarcinoma, and lung cancer (Fig. 2k; Supplementary Fig. 5A–C). These data, in line with a positive role for eIF4E in EMT^24^, establish AXL/MYC/eIF4E as central signaling nodes required for adaptive resistance in *KRAS*-mutant lung cancer cells.

### Deregulation of AXL/eIF4E activates an ER stress-relief UPR survival mechanism

**Fig. 3:**
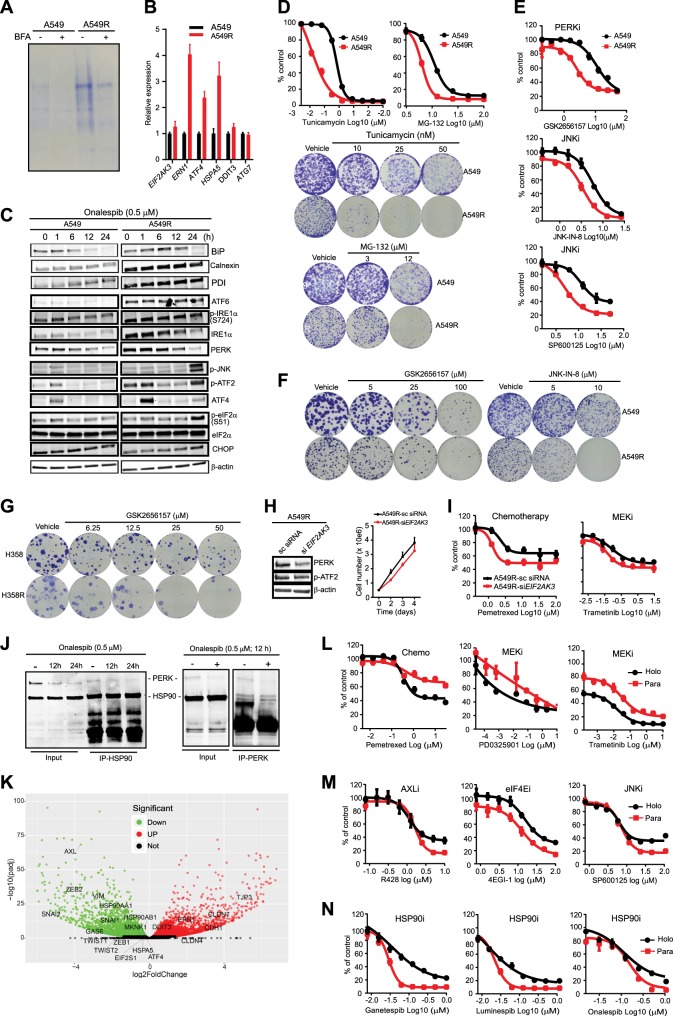
AXL/eIF4E-regulated ER stress-relief UPR underpins drug resistance and intratumor heterogeneity in *KRAS*-mutant lung cancer. **a** A549 and A549R cells without or with brefeldin A (5 µg/ml) treatment (6 h) are profiled for secreted proteins. Shown are representative data of three independent experiments (*n* = 3). **b** Transcriptional quantification of UPR genes in A549R and A549 cells by qRT-PCR. **c** Immunoblots of A549 and A549R cells treated onalespib (0.5 µM) for the indicated time. **d–g** Dose–response curves to tunicamycin (**d**), MG-132 (**d**), and inhibitors to PERK and JNK (**e–g**) assayed on resistant (A549R, H358R) versus parental (A549, H358) cells. Representative results of clonogenic assays are shown to the right. **h**, **i** A549R cells transfected with PERK-specific and control siRNAs were subjected to immunoblotting (**h**) and dose–responsive curves to pemetrexed and trametinib (**i**). Growth curves of the transfected cells are also shown (**h**). **j** Protein lysates (200 μg) prepared from A549R cells treated with onalespib or vehicle were subjected to coimmunoprecipitation with HSP90 and PERK antibodies. Proteins in the precipitates (IP) and the starting material (input) were visualized by western blotting. **k** Volcano plots of transcriptomic profiling (RNAseq) of the A549_holo and A549_para clones. The *X*-axis is the fold-change (log2) of individual genes in the holoclone versus paraclone. Genes significantly downregulated (adjusted *p* value <0.05) in the holo compared to paraclones are shown in green and genes significantly upregulated in the A549_holo versus A549_para are marked in red. Note that *HSP90AA*1 and *HSP90AB1* (encoding HSP90) and the genes involved in EMT, AXL signaling, and the UPR are enriched in the A549_para clone. **l–n** Dose–response curves to MTA and the MEK inhibitors PD0325901 and trametinib (**l**), inhibitors of AXL, eIF4E, and JNK (**m**) and HSP90 (**n**) as determined on A549_holo_clone (black) and A549_para_clone (red) subpopulations. Data are mean ± s.d. of three biological replicates (*n* = 3)

To dissect the effector pathways downstream of AXL/MYC/eIF4E signaling, we conducted an integrative analysis of TCGA data, which significantly linked eIF4E to ER-related functions (Supplementary Fig. 5D–F). We therefore reasoned that deregulation of AXL/eIF4E induces ER stress and activates an ER stress-responsive mechanism. Indeed, The EMT status and *AXL* expression were significantly correlated with the UPR gene signature (Supplementary Fig. 5G, H). Importantly, compared to parental cells, A549R showed a higher magnitude of protein secretion (Fig. 3a), upregulated expression of UPR genes [*HSPA5* (GRP78/BiP), *EIF2AK3* (PERK), *ERN1* (IRE1α), and *ATF4* (ATF4)] (Fig. 3b), increased levels of ER chaperons (BiP, Calnexin, and PDI), ER stress sensors (ATF6, IRE1α, and PERK), and UPR effectors (p-JNK and p-ATF2) (Fig. 3c). In sharp contrast, eIF2α and CHOP that catalyze a malfunctional UPR and induce apoptosis^25,26^ were repressed in A549R (Fig. 3c). Consequently, further perturbations of ER homeostasis by treating with tunicamycin, MG-132, and thapsigargin that induce persistent ER stress preferentially impaired A549R (Fig. 3d; Supplementary Fig. 6A). Importantly, A549R and H358R were particularly susceptible to inhibitors of PERK (GSK2656157), JNK (JNK-IN-8, SP600125), but not of IREα (4µ8c), p38 (LY2228820), or eIF2α (Salubrinal), compared to the parental cells (Fig. 3e–g; Supplementary Fig. 6B). Genetic depletion of PERK reduced p-ATF2 and re-sensitized A549R cells to MTA and trametinib (Fig. 3h, i), whereas it induced an MET in A549 cells (Supplementary Fig. 6C). In line with these results, analyzing gene ontology terms and TCGA data significantly associated *ATF2* with EMT, *PERK* with *ATF2* and *JNK* but not with *MAPK14* (p38) in *KRAS*-mutant lung cancer (Supplementary Fig. 6D–F).

R428 strikingly increased p-eIF2α and CHOP on top of the induction of apoptosis (cleaved caspase 7), indicating that hyperactive AXL/eIF4E signaling is functionally important for PERK/JNK/ATF2-dependent UPR (Fig. 2b). Similarly, *EIF4E* knockdown perturbed the UPR by substantially inducing p-ATF2 and p-eIF2α (Fig. 2h). Thus, deregulation of AXL/eIF4E in drug-resistant cancer cells invokes ER stress, which in turn activates a stress-relief UPR mediated by the PERK/JNK/ATF2 cascade.

### HSP90 controls PERK/JNK/ATF2 integrity and protects from a malfunctional UPR

Next, we sought evidence that connects PERK/JNK/ATF2-dependent UPR with HSP90. Blocking HSP90 function with onalespib profoundly interfered with ER stress responses, measured by dramatic decrease of the PERK and IREα protein levels^27^ (Fig. 3c), and marked inhibition of the HSP90/PERK complex formation (Fig. 3j) in A549R cells. Notably, onalespib treatment enormously upregulated ER stress-induced UPR genes (Supplementary Fig. 7A) and pro-apoptotic UPR effectors (p-eIF2α and CHOP) at 24 h (Fig. 3c), a time coincident with the induction of apoptotic markers (Cl PARP, Cl Cas7) (Fig. 1g). These results indicate that an intact HSP90 is critical for PERK/JNK/ATF2-dependent UPR that is pro-survival and that HSP90 blockage tips the balance from survival towards apoptosis by turning on a malfunctional UPR^25,26^ executed by the eIF2α/CHOP axis. In agreement, the UPR gene signature is significantly correlated with unfavorable clinical outcomes in patients with lung and pan-cancers (Supplementary Fig. 7B–E).

### HSP90/AXL/eIF4E-regulated UPR regulates de novo intratumor heterogeneity of *KRAS*-mutant lung cancer cells

**Fig. 4:**
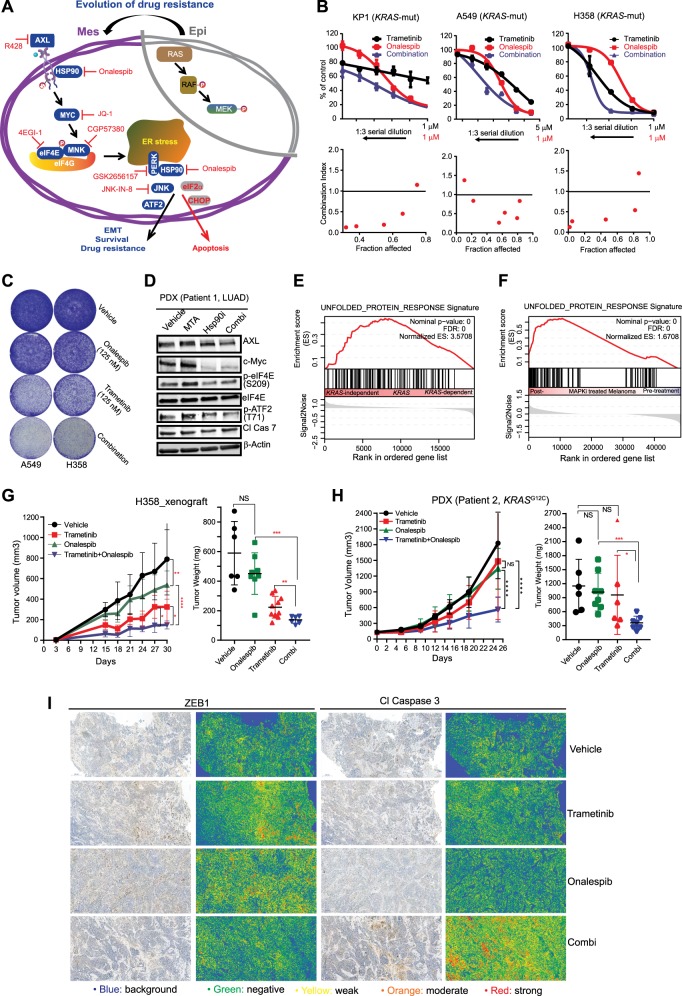
HSP90 antagonism enhances antitumor effects of MTA and trametinib in preclinical cancer models. **a** Schematic overview of acquired vulnerabilities resulting from drug resistance. Epi epithelial cell state, Mes mesenchymal cell state. **b** Growth inhibition of *KRAS*-mutant murine (KP1) and human (A549, H358) lung adenocarcinoma cell lines after treated with trametinib, MTA, and onalespib, alone or in combinations. Each drug was dosed at the indicated concentrations for single treatment or mixed following threefold serial dilutions for combination treatment. Data are mean ± s.d. of three biological replicates (*n* = 3). Plots of fraction affected (Fa) versus combination index (CI) was determined by the CompuSyn software. CI < 1 indicates synergism. **c** HSP90 inhibition enhances trametinib effectiveness by preventing adaptive drug resistance. H358 and A549 cells treated (24 h) with onalespib and trametinib, alone or in combination, were washed with PBS and subjected to further culture for additional 10 days in the absence of the drug. **d** Immunoblots of a lung adenocarcinoma patient (patient 1)-derived xenograft tumors cultured ex vivo and treated with vehicle, pemetrexed (1 µM), and onalespib (0.5 µM), alone or in combination for 24 h. **e**, **f** GSEA of *KRAS*-mutant cancer cell lines (**e**) and matched tumor samples (pre- and post-treatment with MAPK inhibitors) of patients with *BRAF*-mutant melanoma (**f**) show significant enrichment of UPR gene signatures in *KRAS*-independent cell lines (**e**) and in residual melanoma that survived BRAF inhibitors (**f**). The GEO dataset GSE15126 and GSE65185 were used for the analysis. **g**, **h** Volumes of H358 xenografts (**g**) and a *KRAS*-mutant lung cancer patient (patient 2)-derived xenograft model (**h**) treated with vehicle or the indicated drugs. *p < 0.05, **p < 0.01, ****p < 0.0001 by two-way ANOVA. Tumor weights at the end of the treatment are shown to the right. *p < 0.05, **p < 0.01, ****p < 0.0001 by unpaired two-sided *t-*test. **i** Immunohistochemistry (IHC) analysis for EMT (ZEB1) and apoptosis (Cl caspase 3) in H358 xenograft tumors after treated with the indicated drugs. Original overall magnification ×100 (scale bar: 200 μM). Images were taken and processed using CaseViewer software. Both the original IHC slides and the corresponding slides with gradient map visualization were shown. Blue: insignificant (background); green: moderate significant; yellow: significant; orange: more significant; red: most significant

Our models recapitulate tumor progression upon cancer drugs, which might differ from that occurring under pathological circumstances such as de novo intratumor heterogeneity. A549 cells displayed marked heterogeneity^28–30^, evidenced by coexistence of epithelial (holoclone) and mesenchymal (paraclone) subpopulations under standard culture conditions (Supplementary Fig. 8A). Transcriptomic profiling of the A549 holoclone and paraclone^30^ revealed overexpression of epithelial [*CDH1* (E-Cadherin), *TJP3* (tight junction protein ZO-3), *CLDN4/7* (epithelial tight junction proteins Claudin 4/7)], and mesenchymal (*SNAI2*, *ZEB2* and *VIM*) markers, respectively. Notably, numerous genes encoding key components of the resistance pathway, most prominently *GAS6* [encoding growth arrest-specific protein 6 (GAS-6), a cognate ligand of AXL], *AXL* (AXL), *MKNK1* (MNK1), *HSP90AA1*, and *HSP90AB1* that encode the cytosolic isoforms HSP90α and HSP90β, respectively, were expressed at significantly higher levels in A549 paraclones than the holoclone (Fig. 3k). Importantly, the A549 paraclone that displayed heightened resistance to MTA and MEK inhibitors (trametinib, PD0325901) was highly susceptible to inhibitors of AXL, eIF4E, JNK, and HSP90 compared to the A549 holoclone (Fig. 3l–n). Thus, HSP90/AXL/eIF4E-regulated UPR that underpins adaptive resistance to MTA and trametinib also regulates intratumor heterogeneity of *KRAS*-mutant lung tumor cells that occurs under pathological conditions, extrapolating our findings to a generalized principle that governs tumor progression (Fig. 4a).

### HSP90 blockage enhances antitumor effects of MTA and trametinib in lung cancer models

Our finding that HSP90/AXL/eIF4E-regulated UPR fosters tumor evolution and heterogeneity provides a conceptual framework for developing rational therapeutic strategies to treat *KRAS*-mutant lung cancer. As proof of concept, we tested if antagonizing HSP90 enhances antitumor effects of MTA and trametinib. Onalespib strongly synergized with trametinib and MTA in inhibiting the proliferation of *KRAS-*mutant A549, H358, and murine KP cells but not of *KRAS* wild-type H3122 and PC9 cells (Fig. 4b; Supplementary Fig. 8B, C). Consistently, short-term exposure (24 h) to the combined treatment with trametinib and onalespib dramatically suppressed A549 and H358 cell growth to a much greater extent than single drug alone (Fig. 4c). Ex vivo organotypic culture and treatment (24 h) of patient-derived xenograft (PDX) tumors revealed that MTA activated the adaptive resistance mechanism, gauged by marked increase of MYC, p-eIF4E, and p-ATF2, which, however, was effectively counteracted by addition of onalespib (Fig. 4d). In agreement, analyzing transcriptomic data from previous studies^2,15^ revealed that the UPR gene signature was significantly enriched in *KRAS*-mutant cancer cells that underwent an EMT and acquired *KRAS* independence (Fig. 4e) and in residual *BRAF*-mutant melanoma resistant to and relapsed after the concurrent treatment with BRAF and MEK inhibitors (Fig. 4f). These results underscore the ability of MAPK inhibitors to activate the UPR in preclinical models and cancer patients.

Finally, we assessed in vivo efficacy of the combined treatment with trametinib and onalespib, both of which were administrated at doses significantly below clinically achievable levels^9,31^. The combination showed potent antitumor effects in H358 xenografts and a PDX model of *KRAS*-mutant lung cancer without apparent toxicities (Fig. 4g, h; Supplementary Fig. 8D). Importantly, residual tumors after the combination therapy showed augmented apoptosis (Cl Cas3) and reduced proliferation (Ki-67), accompanied by marked attenuation of adaptive properties, e.g., mesenchymal phenotypes (ZEB1), compared to those treated with trametinib alone (Fig. 4i; Supplementary Fig. 8E). These results interrogate our in vitro and ex vivo results and the clinical data of patients, emphasizing the importance to investigate and harness acquired dependency (collateral sensitivity) resulting from tumor evolution and heterogeneity to treat *KRAS*-mutant lung cancer and perhaps other malignancies as well.

## Discussion

In this study, we uncovered that AXL/eIF4E-regulated UPR signaling network, with HSP90 at the helm of the regulatory hierarchy, is an acquired dependency of and confers a selective vulnerability in *KRAS*-mutant lung cancer cells resistant to chemo and anti-MEK cancer drugs. This non-genetic mechanism, unprecedented for MTA resistance^31^ and different from previously reported ones that contribute to adaptive resistance to MEK inhibitors^10–12,17–20^, is clinically relevant in that it underpinned *KRAS*-independent survival of *KRAS*-mutant cancer cells and was activated in patients’ tumors that had acquired resistance to MEK inhibitors. Notably, this collateral sensitivity induced by drug resistance is also applicable to *KRAS*-mutant lung cancer cells that have undergone intratumor heterogeneity under pathological circumstances. Therefore, pharmacological and/or genetic blockage of key nodes of the HSP90/AXL/eIF4E/UPR signaling cascade selectively induces apoptotic cell death of drug-resistant cancer cells, limits tumor heterogeneity in vitro, and precludes the emergence of adaptive phenotypes in vivo.

Our results provide a strong rationale for combining current chemo and anti-MEK cancer drugs with inhibitors of the HSP90/AXL/eIF4E/UPR pathway to treat *KRAS*-mutant lung cancer and perhaps other malignancies for which no effective therapy available for advanced diseases. As preclinical proof of concept, drug combinations of clinically advanced HSP90 inhibitors^32^ with trametinib or MTA potently suppressed cancer cell viability and tumor growth in various tumor models, including a PDX model of *KRAS*-mutant lung adenocarcinoma, validating a long-sought and readily implemented therapy for the most devastating subset of lung cancer. It is also conceivable that HSP90/AXL/eIF4E signaling strength, ER stress magnitudes, and UPR status may stratify subsets of patients with *KRAS*-mutant lung cancer who likely benefit from the combination therapy.

## Materials and methods

### Cell culture and compounds

*KRAS*-mutant (A549, H358) and *KRAS* wild-type (H3122 and PC9) lung cancer cell lines were obtained from American Type Culture Collection (ATCC, Manassas, VA, USA). Murine *KRAS*^*G12D*^; p53^−/−^ cells (KP) were derived from a murine lung adenocarcinoma generated as previously described^33^. Cells were cultured in RPMI-1640 (Sigma-Aldrich, St. Louis, MO, USA), supplemented with 10% fetal bovine seurm (FBS) (Life Technologies, Grand Island, NY, USA) and 1% penicillin/streptomycin (Sigma-Aldrich) at 37 °C in a humid incubator with 5% CO_2_. All cell lines were authenticated by DNA fingerprinting and negative for mycoplasma. Chemical inhibitors used in this study, including those targeting AXL^34^, MYC^35^, eIF4E^36,37^, and PERK^38^, are listed in Supplementary Table 1.

### In vitro modeling of drug resistance

Drug-resistant populations (A549R and H358R) were generated by chronical treatment (6 months) of A549 and H358 cells with stepwise incremental concentrations of MTA (0.1–1.0 µM for A549; 0.5–10 µM for H358) as previously reported^31,39^. Mesenchymal populations (A549_EMT, H358_EMT), the cellular products emanating from mesenchymal reprogramming of A549 and H358 cells, were obtained after 3-month treatment (5 ng/ml for A549; 2.5 ng/ml for H358) with TGF-β1 (AF-100-21C; Peprotech, London, UK).

### Cell viability assay and quantitative analysis of drug synergy

Cells seeded in triplicate at 96-well plates (1000–1500 cells/well) were drugged 24 h later with various compounds as indicated. Cell viability was determined 72 h post-treatment by the Acid Phosphatase Assay Kit (ab83367; Abcam, Cambridge, UK) according to the manufacturer’s protocol. Drug synergism was determined by CompuSyn software, which is based on the median-effect principle and the combination index–isobologram theorem^40^. CompuSyn software generates fraction affected (Fa) and combination index (CI) values. CI < 1.0, synergism; CI = 1.0, additive effects; CI > 1.0, antagonism.

### Clonogenic assay

Cells seeded in triplicate at 10^3^–10^4^ cells/well (six-well plates) were allowed to adhere overnight before treated with various inhibitors or vehicle control for 24 h. The treated cells were subsequently cultured in drug-free medium for 7–14 days depending on growth rates, with culture media replaced every 3–4 days. Surviving cells were then fixed with methanol (1%) and formaldehyde (1%), visualized by staining with crystal violet (0.5% dissolved in 25% methanol). All experiments were performed in three biological replicates and shown were representative experiments.

### Immunoblotting, immunoprecipitation, and immunohistochemistry

Cell lysates were prepared using RIPA buffer (Cell Signaling Technology, Hercules, CA, USA) containing protease inhibitor cocktail and phosphatase inhibitors (Santa Cruz, Dallas, TX, USA). Equal amounts of total proteins were separated by SDS-PAGE (4561033; Bio-Rad) and transferred to nitrocellulose membranes (170-4156; Bio-Rad). After brief incubation with blocking buffer (927-4000; Li-COR Biosciences, Bad Homburg, Germany) at room temperature, the membranes were blotted with primary antibodies (Supplementary Table 2) and anti-rabbit (926–32211) or anti-mouse (926-68020) secondary antibodies (Promega, Madison, WI, USA). Membrane-bound secondary antibodies were visualized by the Odyssey Infrared Imaging System (Li-COR Biosciences).

For immunoprecipitation, precleared cell lysates (200 µg) and monoclonal anti-mouse HSP90 or anti-rabbit PERK antibodies conjugated with Protein G (37478 P) or Protein A agarose beads (9863 P) were mixed by gentle rocking overnight at 4 °C. The beads were washed five times with lysis buffer and bound proteins were eluted by SDS sample buffer. Proteins in the precipitates were analyzed by immunoblotting.

For immunohistochemistry, tissues were fixed in formalin, embedded in paraffin and cut into 4 μm sections. Sections were stained with hematoxylin and eosin and subjected to immunohistochemical staining following standard protocols. Visualization was performed using the Bond Polymer Refine Detection kit (Leica Biosystems, Newcastle, UK) as instructed by the manufacturer. Images were acquired using PANNORAMIC® whole slide scanners and processed using CaseViewer (3DHISTECH Ltd, Budapest, Hungary). In parallel, the staining intensity was shown as blue (background), green (negative), yellow (weak), orange (moderate), and red (strong) using the gradient map visualization plugin within CaseViewer. The following primary antibodies were used: Ki-67 (M7240; Dako, Santa Clara, CA, USA), ZEB1 (HPA027524; Sigma), and cleaved caspase 3 (9664; Cell Signaling Technology).

### qRT-PCR

Total RNA was isolated and purified using RNeasy Mini Kit (74106; Qiagen, Hilden, Germany). Complementary DNA (cDNA) was synthesized by the High capacity cDNA reverse transcription kit (4368814; Applied Biosystems, Foster City, CA, USA) according to the manufacturer’s instructions. Real-time PCR was performed in triplicate on a 7500 Fast Real-Time PCR System (Applied Biosystems) using TaqMan primer/probes (Applied Biosystems): *HSPA5*, Hs00607129_gH; *ERN1*, Hs00980095_m1; *EIF2AK3*, Hs00984003_m1; *ATF4*, Hs00909569_g1; *DDIT3*, Hs00358796_g1; *BBC3*, Hs00248075_m1, and *ATG7*, Hs00893766_m1. *GAPDH* (Hs02786624_g1) and *ACTB* (Hs01060665_g1) were used as endogenous normalization controls.

### Gene silencing by small interfering (siRNA) and short-hairpin RNAs (shRNA)

Cells cultured in triplicate at 50–70% confluency were transfected using SiTran1.0 (TT300001; Origene Technologies, Rockville, MD, USA) according to the manufacturer’s protocol. *EIF4E* and *EIF2AK3* were knocked down by specific pooled siRNA duplexes (SR320018 and SR306267; OriGene Technologies), with control siRNA Duplex as a negative control.

Knockdown of *MYC* was achieved via lentiviral delivery of *MYC* Human shRNA Plasmid Kit (TL311323; OriGene Technologies). A scrambled shRNA was used as a control. Lentiviral particles were generated and cells infected according to the protocol from Broad Institute. The supernatant containing lentiviruses was collected, filtered through 0.45 µm filters, and stored in aliquots at −80 °C, or immediately used to infect recipient cells. After infection, cells were selected in puromycin (1.5 µg/ml) and further passaged in culture for functional assays.

### Secretion assay

5 × 10^6^ cells seeded in 10-cm dishes were cultured in FBS-free media for 6 h, with or without treated with 5 µg/ml Brefeldin A (B6542; Sigma-Aldrich). Proteins in the supernatant were precipitated by trichloroacetic acid, separated by SDS-PAGE and visualized by Coomassie blue staining.

### Mining public databases (TCGA, CCLE, GDSC, and GEO), GSEA, and gene ontology terms analysis

Transcriptomic and reverse phase protein array data of cancer patients were obtained from the Cancer Genome Atlas (TCGA). The gene expression and corresponding survival data were extracted for correlation and prognostic analysis using the corresponding packages in R (“corrplot” and “Hmisc” packages for correlation analysis; “maxstat”, “survival”, and “survminer” packages for prognostic analysis). Analysis of the enriched biological pathway, process, and molecular function from Gene Ontology, as well as gene interaction map, were performed using Funrich software^41^. The expression data and drug sensitivity for cancer cell lines were obtained from the Cancer Cell Line Encyclopedia (CCLE)^42^ and Genomics of Drug Sensitivity in Cancer (GDSC)^43^, respectively. Transcriptomic data of *KRAS*-dependent and -independent cancer cell lines (GSE15126)^2^, matched melanoma pre- and post-treated with MAPK inhibitors (GSE65185)^15^ were downloaded from the Gene Expression Omnibus (GEO) database^44^. Gene set enrichment analysis (GSEA) was performed using GSEA software^45^.

### EMT and UPR gene signatures

The EMT status of tumors and cell lines was determined by the EMT gene signature^18^, scored as the sum of a mesenchymal gene set (*FN1* + *VIM* + *ZEB1* *+* *ZEB2* *+* *TWIST1* *+* *TWIST2* *+* *SNAI1* *+* *SNAI2* *+* *CDH2*) minus that of epithelial genes (*CLDN4* *+* *CLDN7* *+* *TJP3* *+* *MUC1* *+* *CDH1*). The UPR gene signature was calculated using the sum of gene expression of *MMP11*, *FN1*, *METRN*, *BGN*, *SLC7A5*, *SPP1*, *CREB3L1*, *HIST1H1C*, *COL5A2*, *ARNT2*, *COL1A2*, *TMED3*, *MTHFD2*, *SLC2A6*, *IER3* *+* *CDH11*, *CTSD*, *SLC20A1*, *PDIA4*, *COL12A1*, *ECM1*, *MAGED2* and PTRH1 as previously described^46^.

### Patient samples

Experiments with surgically resected tumor specimens from lung cancer patients (patient #1, 57-year-old female, lung adenocarcinoma; patient #2, 67-year-old male, *KRAS*-mutant lung adenocarcinoma) were approved by the institutional review board, with informed consent obtained from all patients as per protocol.

### Animal studies

All mouse experiments approved by ethics committee were performed in accordance with institutional regulations governing animal care. Age- and gender-matched NSG (NOD-*SCID IL2Rγ*^*null*^) mice were used for animal experiments with human cell lines and PDXs. For H358 xenografts, tumor cells mixed with Matrigel (356231; Corning) were subcutaneously inoculated in left and right flanks. For the PDX model, tumor tissues were cut into small pieces (5 µm × 5 µm) and inserted into a subcutaneous pocket. Treatment was initiated when tumors were palpable and drugs were prepared in the following solvent: onalespib (2% DMSO + 30% PEG 300 in H_2_O) and trametinib (4% DMSO in Corn Oil). Trametinib (0.3 mg/kg for H358 xenografts, 0.5 mg/kg for PDX) was administered 5 days/week (p.o.), and onalespib (15 mg/kg for H358 xenografts and 30 mg/kg for PDX) twice/week (i.p.). Tumor size/volume was calculated by the formula: (*D* × *d*^2^)/2, where “*D*” refers to the long tumor diameter and “*d*” the short one.

### Ex vivo organotypic culture

Ex vivo organotypic culture and treatment were processed as previously described^39,47^. In brief, freshly explanted PDX tumors were mounted on agarose, soaked in ice-cold sterile phosphate-buffered saline containing antibiotic/antimycotic, and cut into slices (300–500 μM) by Vibratome VT1200 (Leica Biosystems). Tissue slices were placed in ultra-low attachment plates (3471; Corning Incorporated, Corning, NY, USA) and covered by Dulbecco's modified Eagle's medium medium supplemented with 20% FBS and 1% penicillin/streptomycin solution. After overnight culturing at 37 °C in a humid atmosphere with 5% CO_2_ and 95% air, drug treatment was initiated and lasted for up to 72 h.

### Statistical analysis

Data were presented as mean ± s.d., with the indicated sample size (*n*) representing biological replicates. Data analysis was performed by GraphPad Prism 7 (GraphPad Software, Inc., San Diego, CA, USA). Group size was determined based on preliminary experiments but no statistical method was used to predetermine sample size. Group allocation was performed in a blinded manner. All samples that met proper experimental conditions were included in the analysis. Gene expression and survival data derived from the public database, as well as correlation coefficient (Pearson and Spearman), were analyzed using R (version 3.4.3). For survival analysis, patients were grouped by gene expression, where “high” and “low” expression groups were stratified by the optimal cutoff value. Statistical significance was determined by one-way/two-way analysis of variance (ANOVA), Bonferroni’s multiple comparison test, and Student’s *t*-test using GraphPad Prism 7, unless otherwise indicated. *P* < 0.05 was considered statistically significant.

## Supplementary information

Supplementary Material
