# Supplementary material for: HSP90/AXL/eIF4E-regulated unfolded protein response as an acquired vulnerability in drug-resistant KRAS-mutant lung cancer
Source: Oncogenesis. 2019 Aug 20;8(9):45. doi: 10.1038/s41389-019-0158-7 (PMC6702198; doi:10.1038/s41389-019-0158-7)

Supplementary Information

Supplementary figures and tables

Supplementary Figure 1. Adaptive resistance to MTA and trametinib enforces mesenchymal reprogramming of *KRAS*-mutant lung cancer cells

A-E, Dose-response curves to pemetrexed and trametinib and clonogenic proliferative assay of mesenchymal (A549R, H358R, A549_EMT and H358_EMT) and epithelial (A549, H358) populations. Data were mean ± s.d. of at least three biological replicates. Morphological micrograph (D) and immunoblots for EMT markers (A, C, D) were also shown.

F, Mining TCGA data associates EMT with cancer development in pan-cancers. Multiple pairwise comparisons were performed using 'ggpubr' package in R, with adjusted p-values shown on the graph. The p-value was calculated by one-way ANOVA.

G, Kapler-Meier analysis of a TCGA cohort of patients with solid tumors. Dichotomization of patients was based on the optimal cutoff value of EMT scores across all patients. Survival curves and cumulative hazard rate were analyzed and plotted using R 'survival' and 'survminer' packages. The p-value was calculated using the log-rank test.

Supplementary Figure 2. HSP90 and AXL inhibitors selectively target MTA- and trametinib-resistant *KRAS*-mutant lung cancer cells

A, Dose-response curves to pathway inhibitors assayed in A549R and A549 cells.

B-G, Dose-response curves and clonogenic assay of mesenchymal (A549R, A549_EMT, H358R, H358_EMT) and parental (A549, H358) cells treated with inhibitors of HSP90 (ganetespib, luminespib) or AXL (R428). Data were mean ± s.d. of at least three biological replicates (n=3). ***$p<0.001$ by t-test. Immunoblots of A549 and A549_EMT cells with or without treatment (24 h) with onalespib (5 μM) are shown to the right (E).

Supplementary Figure 3. Drug-resistant *KRAS*-mutant lung cancer cells rely on HSP90 but dispense with MAPK and PI3K/AKT/mTOR pathways

A, Immunoblots of H358, H358R and H358_EMT treated with onalespib (1 μM) for the indicated times.

B, Mining TCGA data from patients with pan-cancers associates *HSP90AA1* with tumor progression, recurrence and metastsis. Multiple pairwise comparisons were performed using 'ggpubr' package in R, with adjusted p-value by one-way ANOVA.

C–E, *HSP90AA1* (encoding HSP90) is associated with poor overall survival and increased recurrence risk in patients with lung adenocarcinoma (C), lung cancer (D), and pan-solid cancer (E). Patients were dichotonized by optimal cutoff value of *HSP90AA1* across all patients using the surv_cutpoint function in R 'maxstat' package. Survival curves and cumulative hazard rate were analyzed and plotted using R 'survival' and 'survminer' packages. The p-value was calculated using the log-rank test.

F, Immunoblots of A549 and A549R cells after treatment with onalespib (0.5 μM) for the indicated times.

Supplementary Figure 4. AXL/MYC/eIF4E are central signaling nodes for drug-resistant *KRAS*-mutant lung cancer cells

A, Protein lysates (200 µg) prepared from A549R cells with or without onalespib treatment were subjected to immunoprecipitation with the AXL antibodies. Proteins in the starting material (input), the precipitates (IP) and the resulting supernatant were analyzed by Western blotting.

B, Gene ontology terms determined on the genes positively correlated with *AXL* based on a TCGA cohort of patients with lung adenocarcinoma. Enriched gene ontology terms for biological process are sorted by $-\log_{10}$ (p-value).

C, Correlation matrix between AXL and EMT-related proteins using R package ´corrplot´. Protein expression data for a TCGA cohort of lung adenocarcinoma patients are downloaded from the Reverse Phase Protein Array database. Positive (in blue) and negative (in red) correlations are shown to the right, with color intensity and the size of the circle proportional to the correlation coefficiency. Non-significant correlation coefficient values are left blank. On the right side of the correlogram, the legend color shows the correlation coefficients and the corresponding colors. p-value < 0.05 is considered significant.

D, Correlation between the *AXL* and EMT signature genes as determined in TCGA cohorts of patients with lung cancer, lung adenocarcinoma and *KRAS*-mutant lung adenocarcinoma. Pearson and Spearman coefficient, as well as the significance (p-value), determined using R software are shown (Cor.test function).

E, F, Kapler-Meier analysis of a TCGA cohort of lung cancer patients (E) and two other lung cancer cohorts from Kapler-Meier Plotter Database (F). Patients were dichotomized by the optimal cutoff value of the *AXL* transcripts across all patients. The p-value is calculated using the log-rank test.

G, Immunoblots of A549R cells transfected with *MYC* shRNA or scrambled shRNAs.

H, Correlations between *MYC* and *AXL, EIF4E* and an EMT gene signature. Gene expression data are downloaded from TCGA cohorts of *KRAS*-mutant lung cancer. Pearson and Spearman coefficients, as well as the significance (p-value), are determined using R software.

Supplementary Figure 5. Hyperactive AXL/eIF4E induces ER stress-responsive UPR

A–C, Prognostic significance of *EIF4E* mRNA (A, B) and eIF4E protein (C) levels in patients with lung adenocarcinoma (A, C) and lung cancer (B). Gene expression data were downloaed from TCGA (A, B) and the Reverse Phase Protein Array database of a lung adenocarcinoma cohort (C). Patients were stratified by the optimal cutoff value of *EIF4E* and eIF4E across all patients using the surv_cutpoint function in the R 'maxstat' package. Survival curves and cumulative hazard rate were plotted and analyzed using R 'survival' and 'survminer' packages. The p-value was calculated using the log-rank test.

D, Gene ontology analysis of genes significantly correlated with *EIF4E* ($p<0.05$) based on a TCGA cohort of patients with lung adenocarcinoma. Enriched gene ontology terms for biological process are sorted by $-\log_{10}$ (p-value) with Funrich software.

E, The five top-ranking genes that positively correlate with *EIF4E* according to (D).

F, The interaction map of the genes that significantly correlate with *EIF4E*.

G, H, Correlations between EMT, UPR and *AXL*. Gene expression data are downloaded from TCGA cohorts of *KRAS*-mutant lung cancer, lung adenocarcinoma and lung cancer. Pearson and Spearman coefficients, as well as the significance (p-value), are determined using R software (Cor.test function).

Supplementary Figure 6. PERK/JNK/ATF2 mediates an ER stress-relief UPR survival pathway in drug-resistant *KRAS*-mutant cancer cells

A, Dose-response curves to thapsigargin and clonogenic assay of A549 and A549R cells. Data were mean ± s.d. of at least three biological replicates (n = 3).

B, Dose-response curves to inhibitors of key UPR signaling nodes (PERK, JNK, IRE1α, p38, eIF2α) measured in drug-resistant (A549R, H358R, H358_EMT) and parental cells. Data were mean ± s.d. of at least three biological replicates (n = 3).

C, Immunoblots of A549 cells transiently transfected with *EIF2AK3* (PERK)-specific or control siRNAs.

D, Gene ontology terms analysis of genes significantly correlated with *ATF2* ($p<0.05$) based on a TCGA cohort of patients with lung adenocarcinoma. Enriched gene ontology terms for biological process are sorted by $-\log_{10}$ (p-value) with Funrich software.

E, F, Correlation analses between *EIF4E, EIF2AK3*, *ATF2* and *MAPK8* gene expression in a TCGA cohort of patients with *KRAS*-mutant lung cancer (n=153). Pearson and Spearman coefficient, as well as the significance, were determined using R software (Cor.test function).

Supplementary Figure 7. HSP90 regulates PERK/JNK/ATF2 integrity and protects from malfunctional UPR

A, qPCR–based transcriptional profiles of UPR genes in onalespib-treated A549 and A549R cells. Data are mean ± s.d. of three biological replicates (n = 3).

B, Ontological significance of the UPR in cancer development and progression as analyzed in a TCGA cohort of pan-cancers. Multiple pairwise comparisons were performed using 'ggpubr' package in R, with adjusted p-values shown on the graph. The p-values were calculated by by one-way ANOVA.

C-E, Kapler-Meier analysis of TCGA cohorts of patients with lung adenocarcinoma (C), lung cancer (D) and pan-cancers (E). Stratification of patients is based on the optimal cutoff value of the UPR signature genes across all patients. Overall survival curves and cumulative hazard rates were analyzed and plotted by using R 'survival' and 'survminer' packages. The p-value is calculated using the log-rank test.

Supplementary Figure 8. HSP90 inhibitors enhance antitumor effects of MTA and trametinib in preclinical tumor models

A, Microscopic graph of naturally existing epithelial (the Holo clone) and mesenchymal (the Para clone) subpopulations within A549 populations.

B, C, Growth inhibition of *KRAS*-mutant KP, A549 (B) and *KRAS*-wt H3122, PC9 (C) lung cancer cells treated with trametinib, MTA and onalespib, alone or in combinations. Each drug was dosed at the indicated concentration for single treatment or mixed at 3-fold serial dilutions for combinations. Data are mean ± s.d. of three biological replicates (n=3). Plots of fraction affected (Fa) versus combination index (CI) was determined by the CompuSyn software. CI < 1 indicates synergism.

D, Mice body weights during the treatment. *p<0.05, **p<0.01 by t-test.

E, IHC analysis (Ki-67) of H358 xenograft tumors after the indicated treatment for 33 days. Original overall magnification x100 (scale bar: 200 μM). Images were taken and processed using CaseViewer software. Both the original IHC slides and the corresponding slides with gradient map visualization were shown. Blue: insignificant (background); Green: moderate significant; Yellow: significant; Orange: more significant; Red: most significant.

Supplementary Table 1. Chemical inhibitors used in this study

Drug name	Manufacturer	Catalog No.	Target
Onalespib (AT13387)	Selleck	S1163	HSP90
Luminespib (AUY-922)	Selleck	S1069	HSP90
Ganetespib (STA-9090)	Selleck	S1159	HSP90
R428 (BGB324)	Selleck	S2841	AXL
AZD4547	Selleck	S2801	FGFR1/2/3
Tepotinib	Selleck	S7067	c-Met
Crizotinib (PF-02341066)	Selleck	S1068	c-Met, ALK, and ROS1
Gefitinib (ZD-1839)	Selleck	S1025	EGFR
Erlotinib	Selleck	S7786	EGFR
AZD8055	Selleck	S1555	mTOR
Everolimus (RAD001)	Selleck	S1120	mTOR (FKBP12)
Rapamycin (Sirolimus)	Selleck	S1039	mTOR
Trametinib (GSK1120212)	Selleck	S2673	MEK1/2
PD0325901	Selleck	S1036	MEK
CGP 57380	Chem Scene	CS-2779	Mnk1
4EGI-1	Selleck	S7369	eIF4E/eIF4G interaction
Tunicamycin	Chem Scene	CS-5779	Glycoprotein synthesis
Thapsigargin	Tocris	1138	Endoplasmic reticulum Ca^{2+}-ATPases
JNK-IN-8	Selleck	S4901	JNK1, JNK2 and JNK3
SP600125	Chem Scene	CS-0196	JNK1, JNK2 and JNK3
Ralimetinib (LY2228820)	Selleck	S1494	p38 MAPK
GSK2656157	Chem Scene	CS-0196	PERK
4μ8C	Chem Scene	CS-6409	IRE1α
MG-132	Selleck	S2619	Proteasome and calpain
Bortezomib (PS-341)	Selleck	S1013	20S proteasome
PU-WS13	Chem Scene	CS-4953	GRP94
Salubrinal	Tocris	2347	eIF2α dephosphorylation
Stattic	Selleck	S7024	STAT3
QNZ (EVP4593)	Selleck	S4902	NF-κB

Supplementary Table 2. Antibodies used in this study

Antibody	Source	Catalog No.
HSP90	Cell Signaling Technology	4874S
HSP90	Santa Cruz Biotechnology	sc-69703
E-Cadherin	Cell Signaling Technology	14472S
Vimentin	Cell Signaling Technology	5741
PARP	Cell Signaling Technology	9532S
Cleaved Caspase-7	Cell Signaling Technology	8438P
β-Actin	Cell Signaling Technology	3700S
AXL	Cell Signaling Technology	4939S
Phospho-AXL (Tyr702) (D12B2)	Cell Signaling Technology	5724S
MNK1 (C4C1)	Cell Signaling Technology	2195S
Phospho-MNK1 (Thr197/202)	Cell Signaling Technology	2111S
Phospho-eIF4E (Ser209)	Cell Signaling Technology	9741S
eIF4E (C46H6)	Cell Signaling Technology	2067P
c-MYC (D3N8F)	Cell Signaling Technology	13987S
PERK (D11A8)	Cell Signaling Technology	5683S
PERK Phospho (Ser713)	BioLegend	649401
Phospho-eIF2α (Ser51)	Cell Signaling Technology	3398S
ATF-4 (D4B8)	Cell Signaling Technology	11815S
CHOP (L63F7)	Cell Signaling Technology	2895P
BiP (C50B12)	Cell Signaling Technology	3177P
Calnexin (C5C9)	Cell Signaling Technology	2679T
PDI (C81H6)	Cell Signaling Technology	3501T
ATF-6 (D4Z8V)	Cell Signaling Technology	65880S
IRE1α (14C10)	Cell Signaling Technology	3294T
phospho-IRE1 alpha (Ser-724)	Novus Biologicals	NB100-2323SS
Phospho-SAPK/JNK (Thr183/Tyr185) (81E11)	Cell Signaling Technology	4668T
Phospho-p38 MAPK (Thr180/Tyr182) (D3F9)	Cell Signaling Technology	4511T
p38 MAPK (D13E1)	Cell Signaling Technology	8690T
Phospho-ATF-2 (Thr71) (11G2)	Cell Signaling Technology	5112S
β-Catenin (D10A8)	Cell Signaling Technology	8480T
Non-phospho (Active) β-Catenin (Ser33/37/Thr41) (D13A1)	Cell Signaling Technology	8814S
Ki-67	Dako	M7240
ZEB1	Sigma	HPA027524
Cleaved Caspase 3	Cell Signaling Technology	9664
800CW Donkey anti-Rabbit IgG (H + L) Secondary Antibody	LI-COR Biosciences	925-32213
800CW Goat anti-Mouse IgG (H + L) Secondary Antibody	LI-COR Biosciences	926-32210

Supplemental Fig. 1

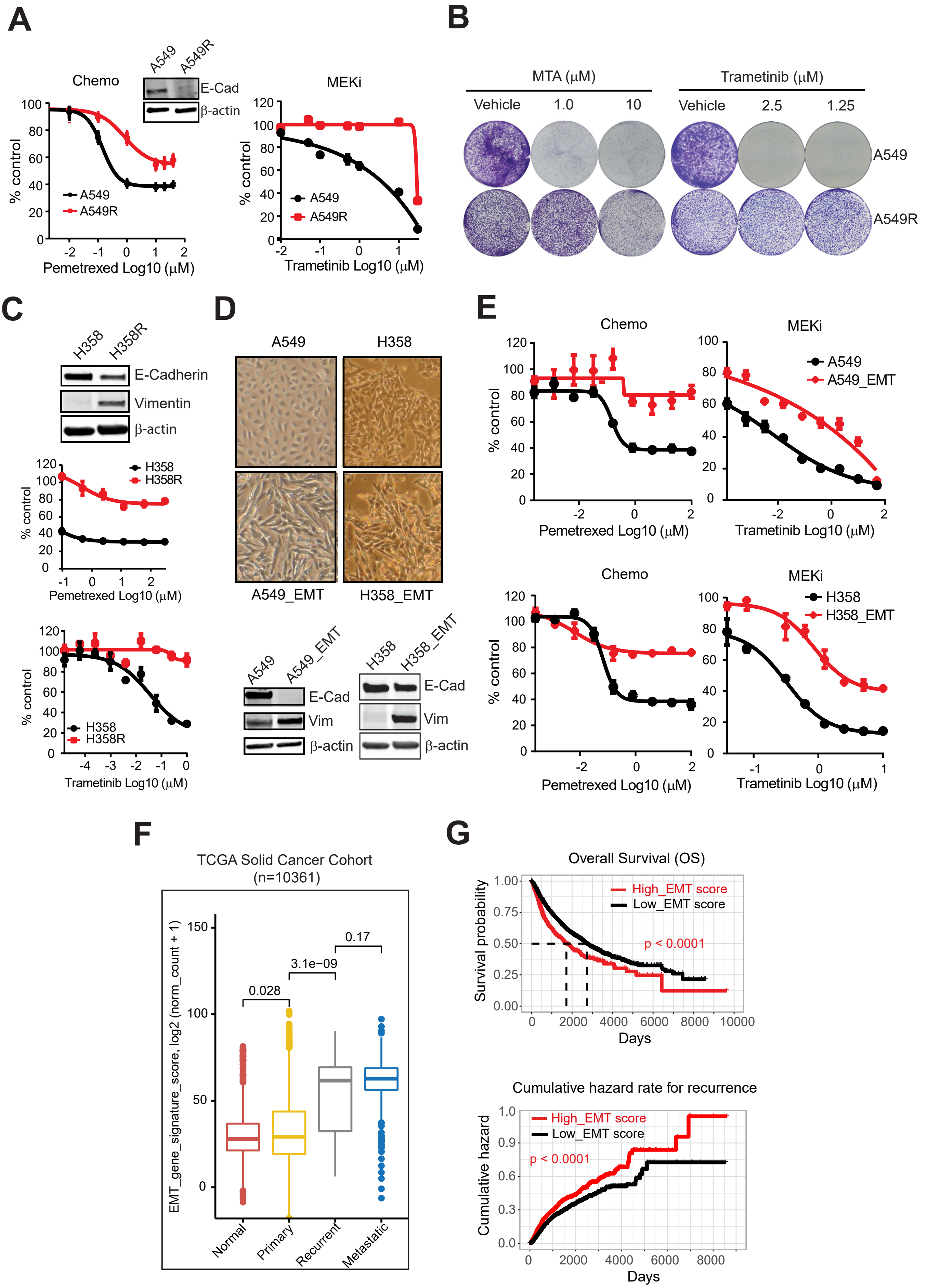

Supplemental Fig. 2

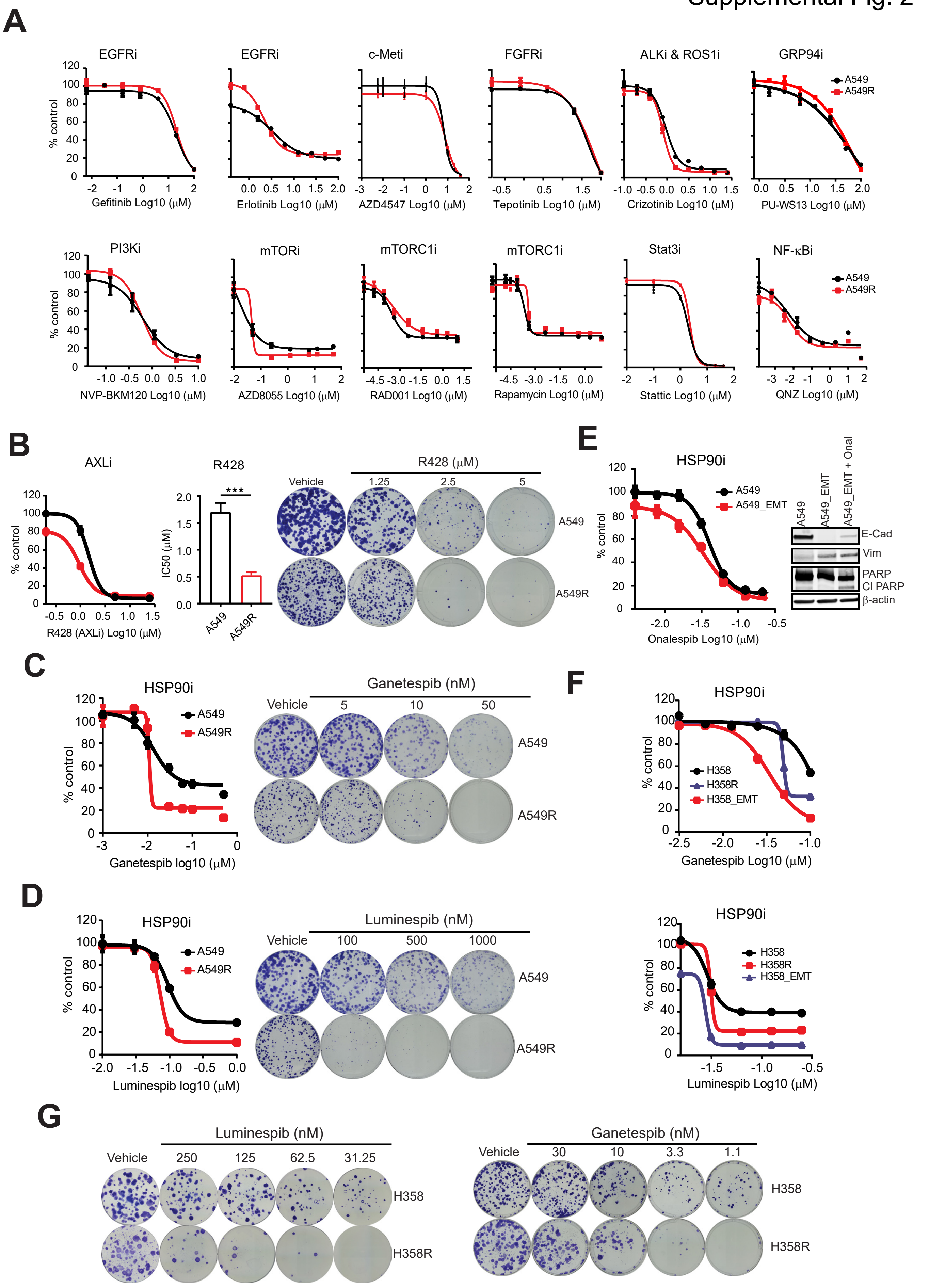

Supplemental Fig. 3

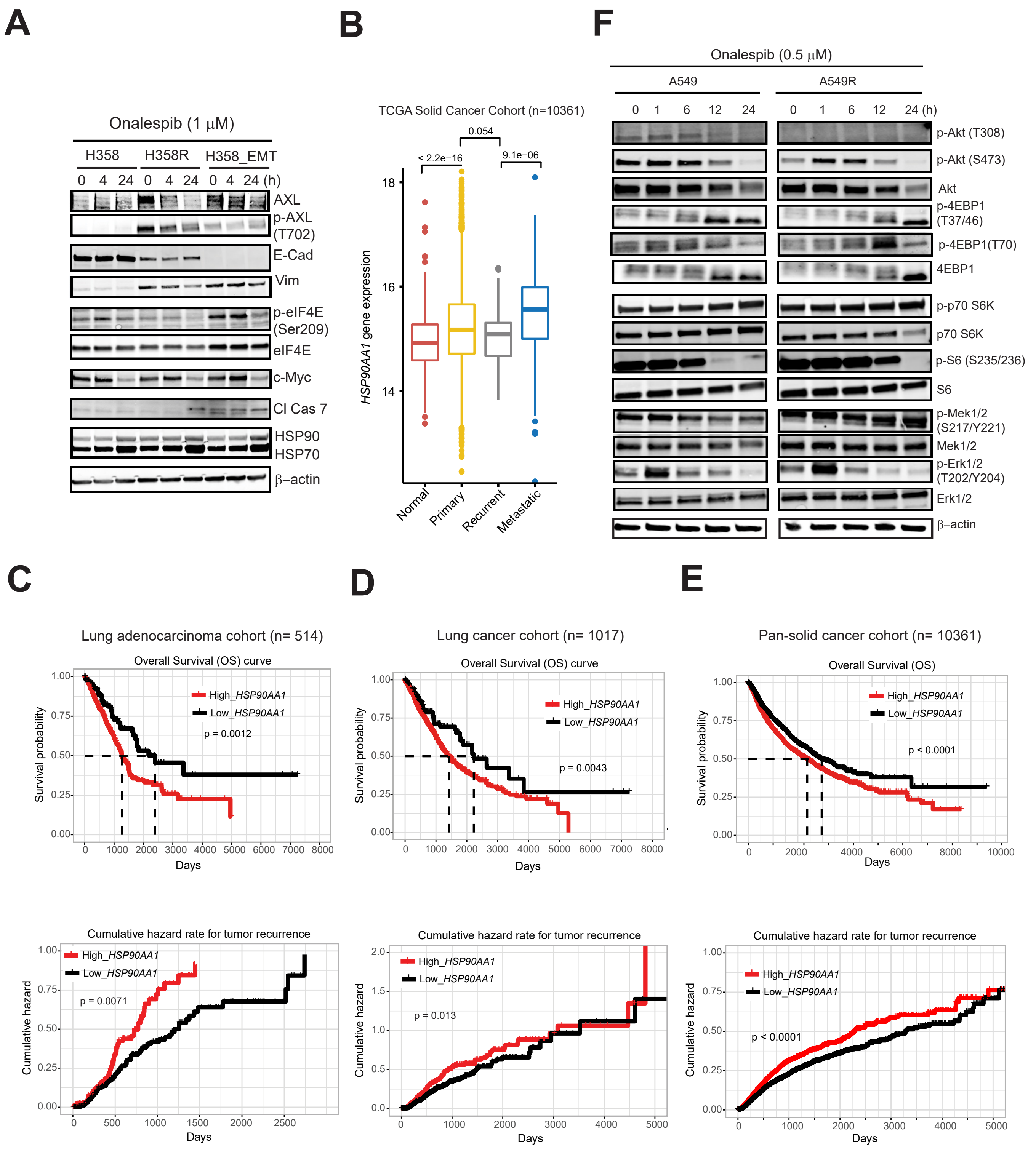

Supplemental Fig. 4

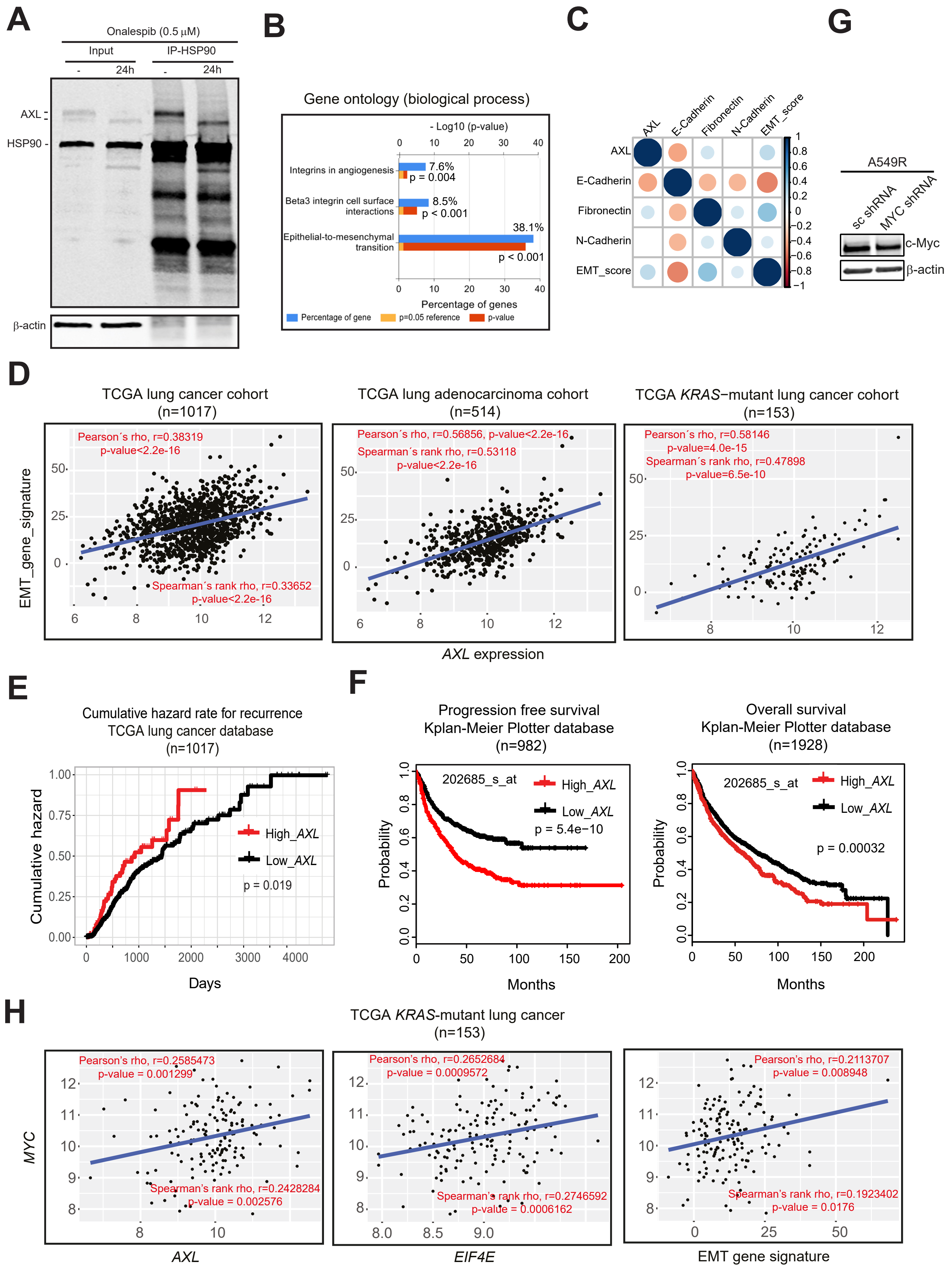

Supplemental Fig.5

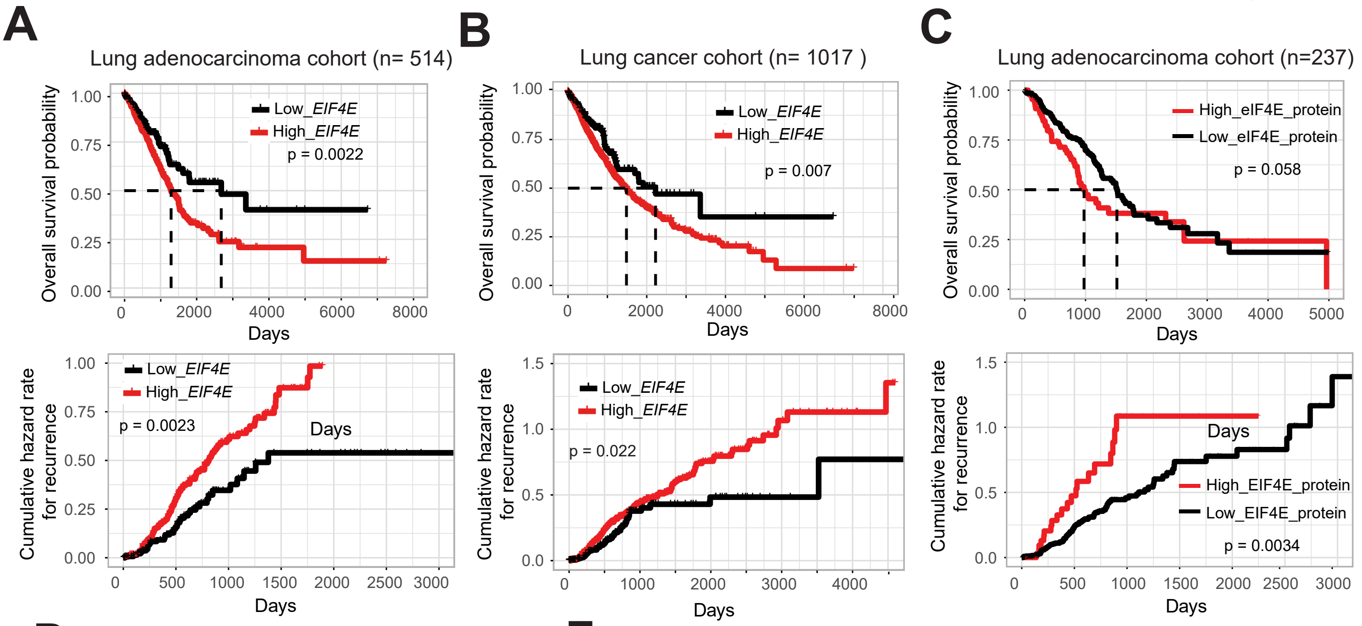

D

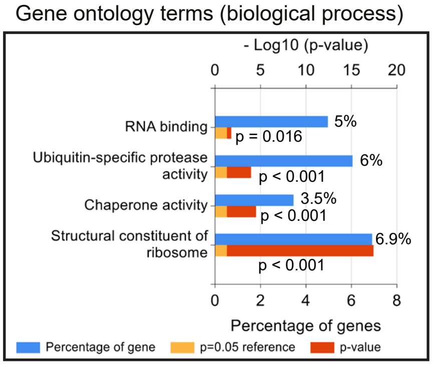

E

Rank	Gene	Pearson r	Function
1	*CISD2*	0.72	ER calcium homeostasis
2	*UBE2D3*	0.7	Ubiquitination
3	*METAP1*	0.69	Methionyl aminopeptidase
4	*MAD2L1*	0.67	Mitosis
5	*COPS4*	0.66	Ubiquitination

F

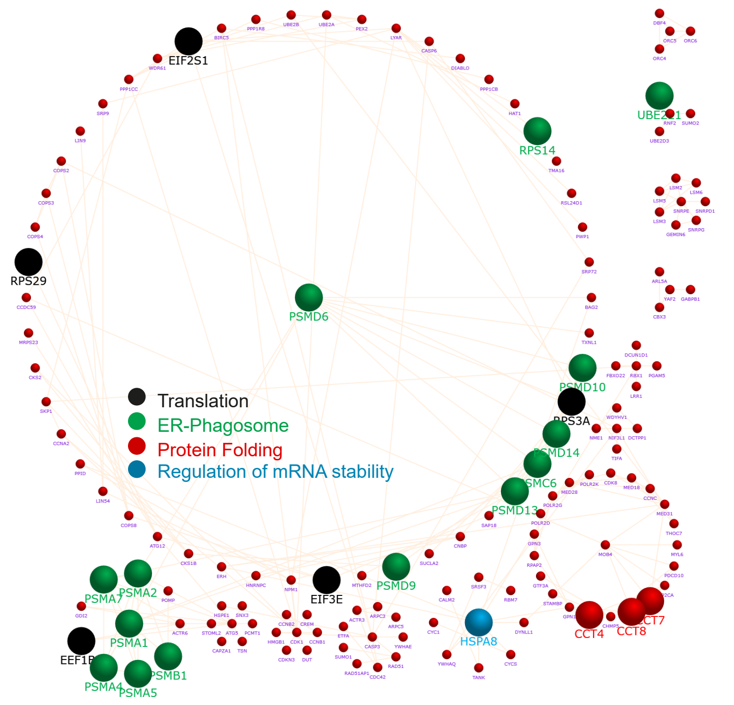

G

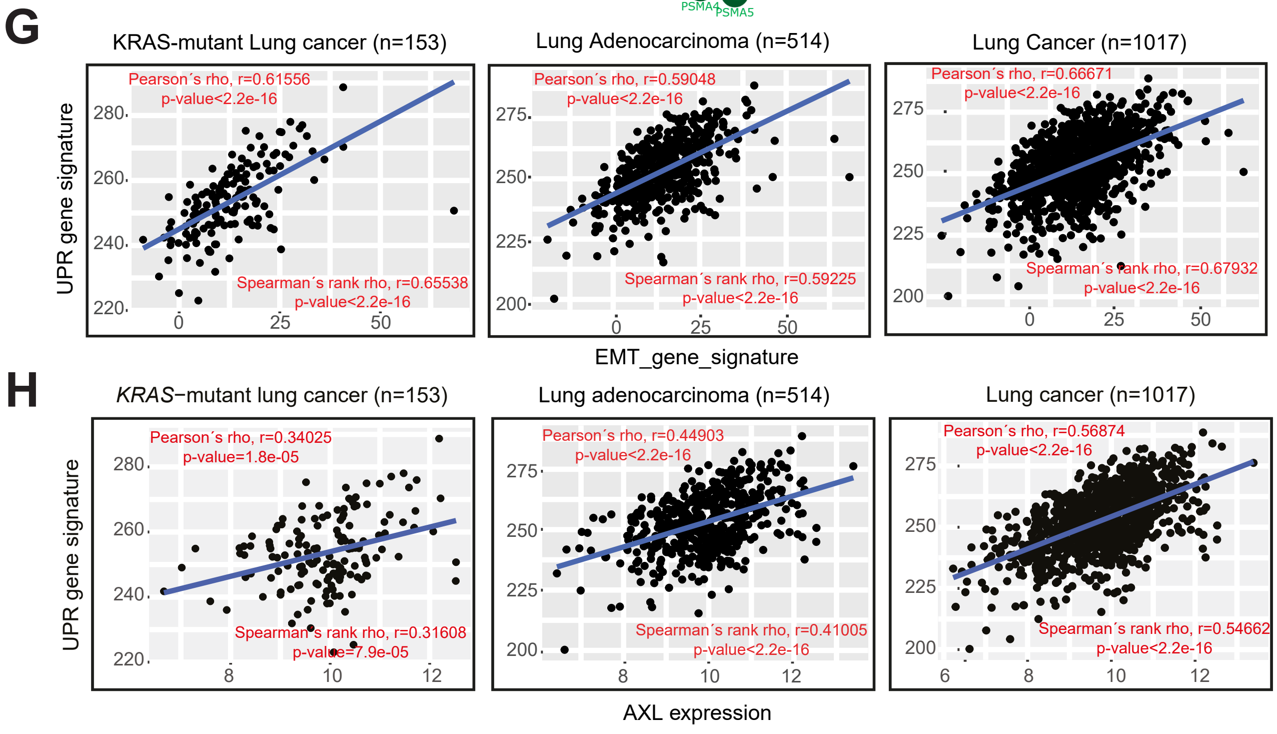

Supplemental Fig. 6

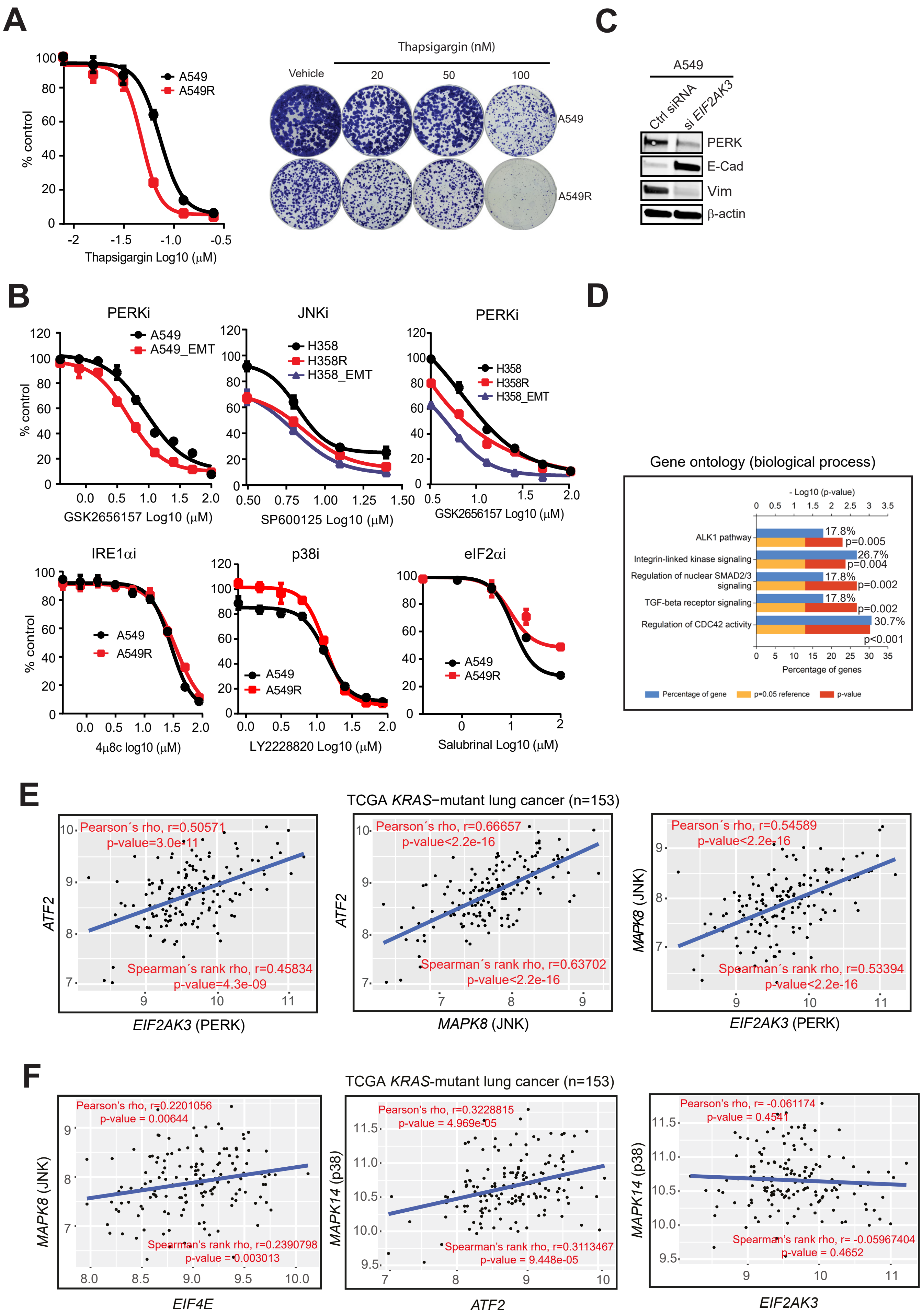

Supplemental Fig. 7

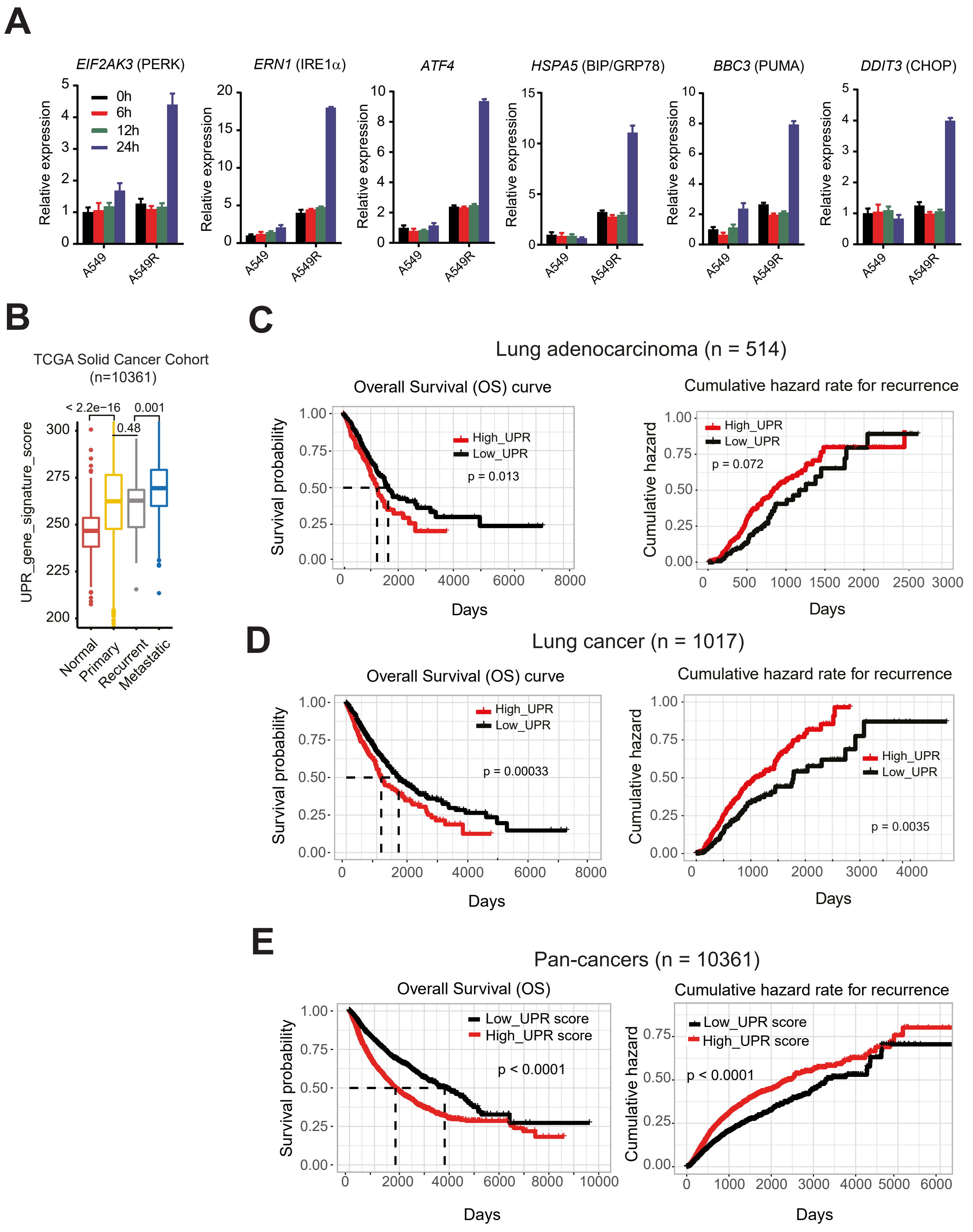

Supplemental Fig. 8

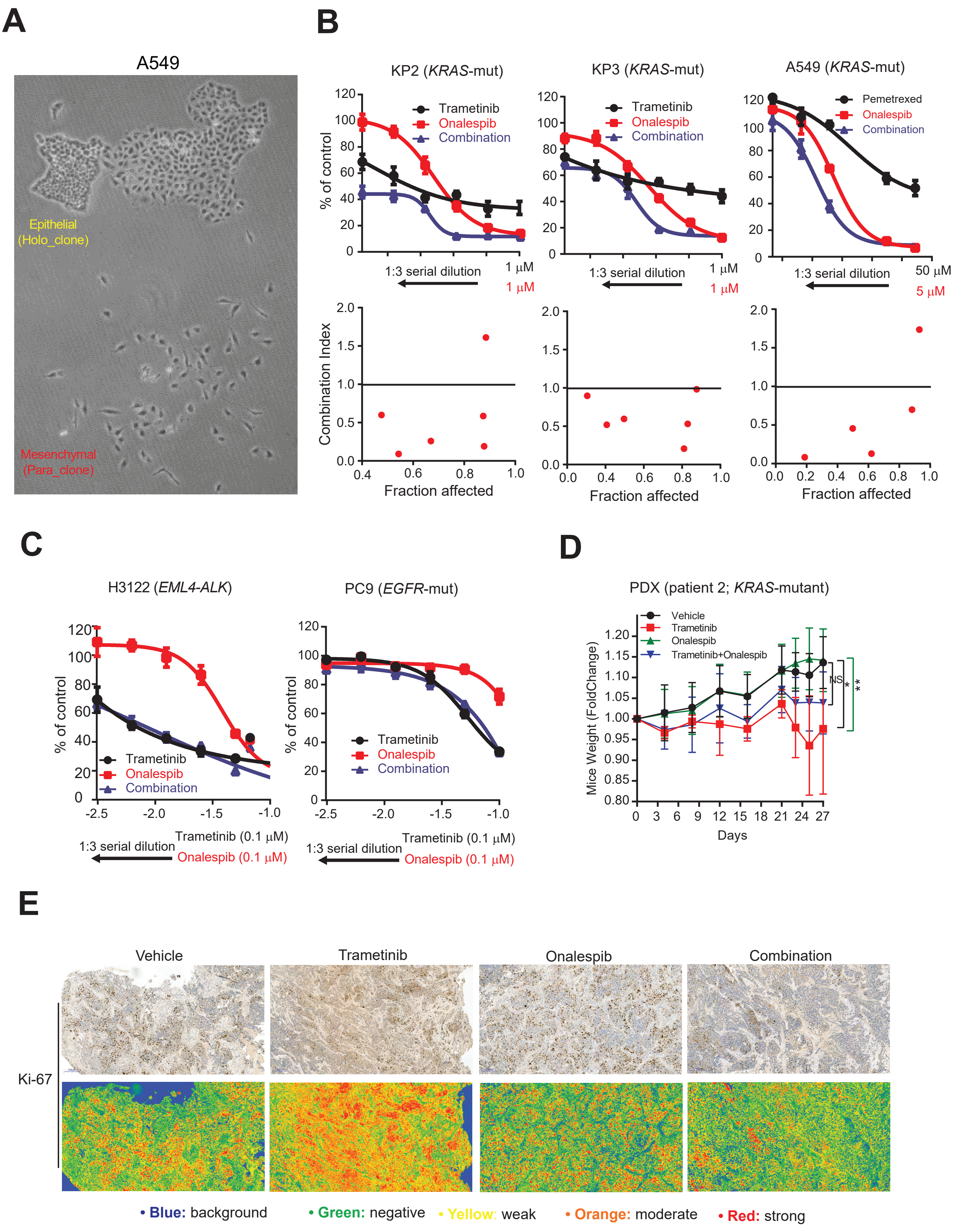

Supplement: Supplementary file 1 — Supplementary Material [file 41389_2019_158_MOESM1_ESM.pdf]
